# Genetic and Clinical Features of Medullary Thyroid Carcinoma: The Experience of a Single Center in Costa Rica

**DOI:** 10.1155/2016/9637173

**Published:** 2016-11-27

**Authors:** Javier Calvo, Gabriel Torrealba, Adriana Sáenz, Carlos Santamaría, Estela Morera, Silvia Alvarado, Yolanda Roa, Michelle González

**Affiliations:** ^1^Division of Endocrinology, Department of Internal Medicine, Hospital Rafael Ángel Calderón Guardia, San José, Costa Rica; ^2^Neuromodulation Center, Spaulding Rehabilitation Hospital, Harvard Medical School, Boston, MA, USA; ^3^Laboratory of Molecular Biology, National Children Hospital, San José, Costa Rica; ^4^Department of Pathology, Hospital Rafael Ángel Calderón Guardia, San José, Costa Rica

## Abstract

*Background*. Activating mutations in the* RET* gene leads to medullary thyroid carcinoma (MTC). Guidelines encourage performing* RET* analysis in subjects with hereditary and sporadic disease.* Materials and Methods*.* Design*. Observational, case series report study.* Patients*. Subjects diagnosed with MTC, with a thyroidectomy performed in a single center in Costa Rica between the years 2006 and 2015.* Diagnosis and Follow-Up*. Pre- and postoperative calcitonin,* RET* mutation, and neck ultrasound and tomography were obtained.* Results*. 21 subjects with histological diagnosis of MTC were followed up. The average age at diagnosis was 52.0 ± 15.7 years. The preoperative mean value of calcitonin was 1340 ± 665 pg/mL. Evidence of* RET* mutation was found in 26.3% of the patients, with only 2 of them grouped in the same kindred. We found statistically significant differences in mean ages between mutated (38.4 ± 20.2 y) versus nonmutated* RET gene* (54.6 ± 11.8 y, *p* = 0.04). There were no significant differences regarding tumor size, metastases, and surgical reintervention.* Conclusions*. We report the results of* RET* mutation analysis in subjects with MTC in a single center of Costa Rica. The availability of this tool increases the probability of identifying familial MTC, with the benefit of detecting affected subjects and their relatives at an earlier age.

## 1. Introduction

Medullary thyroid carcinoma (MTC) is a rare neuroendocrine tumor that accounts for only 3% to 5% of all thyroid gland cancers. However, this neoplastic disorder is one of the best-characterized solid tumors in terms of its pathologic, biochemical, molecular, and genetic properties [1]. Approximately 75% of the cases will present in a sporadic form and the remainder will show a hereditary pattern [2]. A germ-line activating mutation in the* RET* (*REarranged during Transfection*) protooncogene is reported in nearly all cases of hereditary forms of MTC associated with multiple endocrine neoplasia type 2 (MEN 2), and a somatic* RET* mutation can be present in up to 50% of sporadic forms of MTC [3, 4].

The* RET* gene plays an important role in cell signaling in neural tissue, especially during the early stages of development. Mutations in this gene lead to abnormalities in cell proliferation and differentiation of tissues that are derived from neural crest cells. The C-cells of the thyroid and the chromaffin cells in the adrenal medulla are both affected, resulting in MTC and pheochromocytoma, respectively [5]. Twenty years ago, the groundbreaking discovery that an activating* RET* mutation is present in subjects with MTC and MEN 2 opened a new chapter in the era of personalized medicine and genetic association studies [6]. Currently, screening of the* RET* gene is widely applied for the detection of several inherited diseases, not only for the identification of gene carriers when MTC is diagnosed, but also to serve as a preclinical diagnosis in their families [7].

According to the American Thyroid Association (ATA), all MTC cases, whether they are hereditary or sporadic, should have genetic counseling and direct DNA analysis in order to detect mutations in the* RET *allele [8]. If a germ-line* RET *mutation is found, the patient's first-degree relatives should be provided with genetic counseling and testing. Although a change in the genetic sequence of the* RET* gene is considered to be the cause of the MEN 2 syndrome, the mutation must be complemented with the clinical expression of the disease within a family member. This requires that at least two affected individuals present with a familial MTC, MEN 2A or MEN 2B phenotype [9].

Genetic assessments for germ-line* RET* mutations in patients with MTC and MEN 2 have assisted endocrinologists to provide patients, as well as their relatives, with a more accurate diagnosis. Here we present the first Costa Rican population report of MTC cases that include a* RET* mutation analysis.

## 2. Materials and Methods

### 2.1. Patients

In this study we studied a total of 21 subjects from a single tertiary care center from San José, Costa Rica, with a diagnosis of MTC, who had a total thyroidectomy. Patients were surgically treated between the years 2006 and 2015 and molecular blood analysis for* RET* gene mutation was performed in 19 of the cases. This study was approved by the Ethics Committee of Hospital Dr. Rafael Ángel Calderón Guardia.

### 2.2. Calcitonin Measurement

Plasma calcitonin was measured by immunoradiometric assay, using the IMMULITE 1000 Immunoassay System (Siemens®, Germany), with a normal value established as less than 2 pg/mL. Blood samples were collected before the surgery, in the immediate postoperative period, and at least every six months in the follow-up assessments.

### 2.3. Screening for* RET* Mutation, DNA Extraction, and PCR Amplification

Mutational analysis of* RET* gene was carried out by using standard PCR and Sanger sequencing protocols. Briefly, peripheral blood from MTC patients was collected and DNA was automatically extracted by using MagNA Pure 2.0 (Roche Diagnostics®, Switzerland) platform. PCR was performed with a High Fidelity PCR kit (Roche Diagnostics) following protocol according to Jindřichová et al. [10]: initial denaturation at 95°C for 10 min followed by 40 cycles (of denaturation at 95°C for 30 s, annealing at optimized temperature (60–68°C) for 30 sec, and elongation at 72°C for 1 min) and final elongation at 72°C for 10 min. The PCR products were evaluated with a Qiaxell system (Qiagen Company®, Hilden, Germany) and mutations were assessed through Sanger sequencing in a 3500 Genetic Analyzer (Life Technologies®, California, United States) by using BigDye 3.1 chemistry.

### 2.4. Surgical Treatment and Follow-Up

In most of the cases, the surgical treatment consisted of a total thyroidectomy; however two patients only underwent lobectomy due to a prior removal of the contralateral lobe. In the patients where a fine-needle aspiration (FNA) report documented a medullary carcinoma, a bilateral dissection of the central neck compartment in conjunction with a removal of the lymph nodes was performed. If ultrasound evaluation documented suspicious metastatic lesions on cervical lateral lymph node compartments, the surgeon performed an ipsilateral neck dissection. All of the patients were followed up postoperatively for at least six months at the Endocrinology Unit of our hospital. Follow-up assessments consisted of clinical examination, calcitonin measurements, and an ultrasound or CT scan of the neck when needed.

### 2.5. Histopathology

Thyroid gland and lymph nodes were studied using standard histopathological techniques, as well as immunohistochemical stains for thyroglobulin, synaptophysin, and calcitonin.

### 2.6. Statistical Analysis

Statistical analysis was performed using SPSS statistics software (version 22, SPSS Inc., Chicago, IL). Continuous variables were described with mean or median and compared with *t*-test or Mann–Whitney test when appropriate. We used Fisher's exact test for comparisons of categorical variables frequencies. Values of *p* < 0.05 were considered statistically significant.

## 3. Results

In this series of cases, we included a total of 21 subjects with a pathologic diagnosis of medullary thyroid carcinoma. Most of the patients were women (81%, 17/21) and the average age at diagnosis was 52.0 ± 15.7 years. A familial antecedent of MTC was found only in two cases; nonetheless the rest of the patients had no clear knowledge of their family disease records. Remarkably, other diagnoses of* RET* unrelated tumors were found in 23.8% (5/21) of the cases, accounting for a variable tumor origin which was comprised of melanoma, papillary thyroid, parathyroid, gastric, colorectal, breast, and basal cell carcinoma.

In the preoperative evaluation, all of the patients underwent FNA biopsy but only 55% (11/21) of the subjects had the correct medullary carcinoma diagnosis on the biopsy report. A follicular neoplasm was the most frequent report, in 40% (4/10) of the misdiagnosed patients. As a result, less than half of the cases were assessed with a preoperative calcitonin measurement. However, of those who were sampled, we estimated a calcitonin mean value of 1340 ± 665 pg/mL.

We found a germ-line* RET* mutation in 26.3% (5/19) of patients that were evaluated with genetic testing (see Table 1). Patient 5 was diagnosed with MEN 2B. He first presented with a medullary thyroid carcinoma and had surgery when he was 10 years old. During puberty, he developed an evident marfanoid habitus. After the surgical intervention, his calcitonin levels remained stable (less than 300 pg/mL). To date, he has not shown any radiological evidence of recurrence and a pheochromocytoma has not been documented. Alternatively, Patient 2 was diagnosed with MEN 2A. Her first detected tumor was the MTC at 53 years of age. During postsurgical follow-up, a left adrenal mass was documented and confirmed to be a pheochromocytoma after resection. During her follow-up, she had local, bone, and mediastinal recurrences of her MTC, requiring multiple surgeries and radiotherapy.

Patients with a* RET* gene mutation were of a statistically significant younger age at diagnosis (38.4 ± 20.2 years), when compared to patients without these variations (54.6 ± 11.8 years) (see Table 2). Metastases affecting cervical lymph nodes were similar in both groups: 60% (3/5) in the* RET* positive group and 57% (8/14) in the* RET* negative group (*p* = 0.66). A surgical reintervention was required in 40% (2/5) of the patients with the* RET* mutation compared to 35.7% (5/14) of the patients without these genetic variants (*p* = 0.76). There were no statistically significant differences regarding tumor diameter, frequency of vascular invasion, or distant metastases, among the groups compared.

## 4. Discussion

This is the first clinical characterization of patients with MTC obtained from our center that included a genetic screening. After the implementation of a* RET* mutation screening program, we were able to identify germ-line mutations in 26.9% of patients with MTC. This frequency of* RET *mutations is similar to data reported in other case series [11, 12]. The presence of a considerable amount of hereditary tumors among our patients, in addition to the presence of germ-line* RET* mutations in up to 14.9% of patients with sporadic MTC [13], confirmed the need for a routine genetic screening as recommended by ATA guidelines.

Patients‘ mean age of diagnosis was 52.0 ± 15.7 years, which is similar to what has been reported elsewhere [8]. The proportion of sporadic and hereditary tumors found was also consistent with other case series [11]. In our sample we had more women than men, in a ratio of 17 : 4 (F : M), which is different from what has been previously reported [1, 8]. The ratio of women to men found in this study might be associated with the fact that women use the Costa Rican national health system more frequently than men, regardless of the fact that the access to health services is the same for both genders [14].

In this study, fine-needle cytology was only accurate reporting a MTC diagnosis in 55% of the patients. It is known that MTC has a variable appearance in aspiration cytology, which often results in a doubtful diagnosis. In that sense, it is recommended that immunohistochemical studies (i.e., calcitonin or chromogranin) should be used in order to improve the cytological evaluation [15]. Since calcitonin measures were not routinely used it could explain our high percentage of misdiagnosis of MTC. This results in an incomplete preoperative assessment without preoperative calcitonin values, which could lead to possible errors when choosing the best surgical procedure for the patient. Furthermore, it has been reported that relying on only FNA biopsy in sporadic MTC cases could negatively impact patient's initial surgical management, possibly misguiding towards a total thyroidectomy instead of a total thyroidectomy plus central neck dissection procedure when needed [16].

We found that patients who harbored germ-line mutations in their* RET* gene were, on average, 16 years of age younger at diagnosis, when compared to those without these genetic variants. This observation reported here, and in several other sources [6, 11], could be explained by two mainstay reasons. First, during the diagnostic process of an extended screening method for* RET* germ-line mutations, it is easier to find patients' relatives at an earlier age who are commonly in a preclinical stage of the disease [12]. Second, the highest risk mutations (Class D, according to the ATA risk classification) are predominantly present in patients who manifest the disease during the pediatric age [17].

In our study, we found four different germ-line* RET* mutations. According to ATA guidelines [8], one of these was classified as a “highest risk” mutation in one patient (M918T) and the rest were considered as “moderate risk” mutations in four other subjects (V804M and L790F twice and C611R). MTC aggressiveness is usually related to the clinical presentation and the type of* RET* mutation, both in sporadic and in hereditary forms. Particularly in hereditary cases, there is a known association between the* RET* mutation, its phenotype, and the prognosis [11]. According to this,* RET* mutation may determine when the optimal age would be to perform surgery on the patient and whether this procedure may require an extension for lymph node resection in addition to the thyroidectomy [8]. On the other hand, the presence of a* RET* oncogene somatic mutation in sporadic MTC has been associated with both regional and distant metastases, with a higher mortality and a larger tumor size [3].

The presence of germ-line* RET* mutations points towards hereditary forms of MTC [18]. However, it was interesting that Patient 1 had a clinical diagnosis of an apparent sporadic MTC but was also found to have a germ-line mutation (V804M), a situation that has been reported elsewhere [19]. This emphasizes the importance of having routine molecular* RET* analysis in all MTC patients underscoring the fact that there is not always enough criteria to differentiate between a sporadic and a hereditary MTC, even with the clinical presentation, diagnostic laboratory tests, and histologic findings.

Codon 611 (exon 10) of the* RET *gene encodes for an extracellular cysteine-rich domain and is frequently affected with mutations associated both with hereditary MTC and with classical MEN 2A [11]. Patients with these mutations should be screened for familial MTC at 5 years of age and for hyperparathyroidism and pheochromocytoma starting at 16 years of age [8]. We found a C611R mutation in Patient 2, and although she was older when she was diagnosed both with the MTC and with the pheochromocytoma, this mutation still correlated with the typically reported aggressive biological behavior described [11]. Her case showed evidence of distant metastases in bone, mediastinum, and an extensive lymph node compromise.

Patients 3 and 4 were a mother and daughter who both harbored a L790F mutation. They also had a lesion confined to the thyroid gland but none presented with metastases during follow-up. Given the fact that genetic variants in codon 790 are known to have incomplete penetrance [20], patients with this particular mutation could not develop a MTC. Although both of them had the same mutation, the daughter had earlier manifestations and was therefore assessed and diagnosed soon after her mother was treated.

Subjects with MEN 2B have either MTC or C-cell hyperplasia with typical phenotypic features such as marfanoid body habitus, mucosal neuroma, and intestinal ganglioneuromatosis, with or without PHEO [9]. Previous large published series have reported a low frequency of MTC related to MEN 2B syndrome, finding only 6.7% of CMT cases in 38 years of experience [21]. In our sample, only Patient 5 was diagnosed with the M918T point germinal mutation on exon 16 of the* RET *gene. He had a surgical intervention at 10 years of age and has not shown recurrence of MTC after 18 years of follow-up. Similarly, the majority of MEN 2B patients presented clinical manifestations in the second decade of life and needed to have a total thyroidectomy when they were initially diagnosed [8]. Particularly in this patient, the same mutation was not detected in his mother nor had a history of medullary carcinoma been detected in his paternal or maternal relatives, suggesting that this mutation occurred* de novo*, which is a relatively common finding, as approximately 75% of MEN 2B patients have de novo* RET* mutations [22].

The experience over time with* RET* germ-line mutations has also been applied to the analysis of somatic mutations in MTC tissue samples [23]. This has been a growing field in recent years being a feasible test that can be performed in patients (like the 28.6% (4/14) of our sample) who did not harbor a germ-line* RET* mutation but developed distant metastases or recurrences that are not completely resectable. No specific recommendations have been provided by current guidelines regarding the need to investigate somatic mutations in sporadic MTC. However, the evidence on this topic is increasing and is particularly important in aggressive metastatic tumors. A recent publication concerning* RET, RAS, TP53, BRAF, AKT, or PIK3C *somatic mutation testing in 70 cases of advanced and metastatic CMT showed a somatic mutation in 91.4% of the cases assessed. Among the mutated cases,* RET* mutations (mainly M918T) were highly prevalent. However, in a sufficient number of cases, more than one* RET* somatic mutation could be found in the same tumorous tissue, and the subjects with these characteristics were associated with a worse outcome [24].

Reference centers now have long experience in* RET* mutations screening and, after 20 years, some relevant findings have been published. Although it is not possible to estimate the real prevalence of these mutations, we have a clear idea of the distribution regarding the most frequent genetic defects related to familiar MTC, with the V804M point mutation being the most common identified variation [17]. Additionally, besides the impact that this type of genetic evaluation may have on the patients themselves, it is of paramount importance that relatives are screened in order to identify mutated and nonmutated family members. This may give their caring physicians the ability to definitively discriminate patients who should start a clinical and biochemical evaluation program from those who should not, especially in those cases where prognosis would change with an early treatment. This early diagnosis and intervention can increase the possibility for these patients to be definitively cured [17].

This case series report has several limitations. First, it was carried out in a single center and we had a small number of subjects. One reason for this issue was that part of the oncologic group of physicians was not aware of the genetic screening of patients with MTC; therefore many of these patients were not included in the trial initially because they did not have molecular evaluation. Second, because the assessments of the patients were not standardized according to their* RET* mutation profile, we have incomplete data regarding preoperative and postoperative follow-up evaluations. Especially concerning was the lack of data from key laboratory studies (i.e., calcitonin) that were not obtained from our patients.

## 5. Conclusions

We reported the first clinical characterization of patients with MTC with their respective* RET* mutation status, from a single center in Costa Rica. We were also able to identify the most prevalent* RET* mutations, in our small group of patients. A germ-line* RET* mutation was found in 26% of the subjects. Patients with these mutations had a younger age at diagnosis; however, we could not establish significant differences regarding the biologic behavior of the tumors, when compared to patients without these variants. We know this is the first step towards establishing a better clinical MTC evaluation in our center, following ATA guidelines. We also know that routine genetic testing of MTC patients will help us assess the risk for subjects and their relatives for MTC related diseases. This should result in a higher probability of identifying new familial MTC, as part of MEN 2, and, even more importantly, providing an earlier diagnosis to asymptomatic subjects.

## Figures and Tables

**Table 1:** Characteristics of patients with a positive germ-line *RET* mutation and MTC.

Patient number	Age at dx	Exon	Codon	Amino acid change	ATA risk	Clinical diagnosis
1	52	14	804	V/M	A, moderate	Sporadic MTC
2	53	10	611	C/R	B, moderate	MEN 2A: PHEO and MTC
3	24	13	790	L/F	A, moderate	FMTC
4	53	13	790	L/F	A, moderate	FMTC
5	10	16	918	M/T	D, highest	MEN 2B: marfanoid habitus and MTC

ATA: American Thyroid Association, MTC: medullary thyroid carcinoma, FMTC: familial MTC, MEN 2: multiple endocrine neoplasia type 2, and PHEO: pheochromocytoma.

**Table 2:** Comparison of neoplastic behaviors in patients with MTC according to *RET* mutation status.

*RET* mutation (*n*)	Positive (5)	Negative (14)	*p*
Age at diagnosis (years)	38.4 ± 20.2	54.6 ± 11.8	**0.04**
Tumor diameter (cm)	1.48 ± 1.1	2.37 ± 1.6	0.61
Vascular invasion	20%	28.6%	0.71
Nodal metastases at surgery	60%	57%	0.66
Distant metastases	20%	14%	0.87
Surgical reintervention because of MTC recurrence	40%	35.7%	0.76

*RET*: REarranged during Transfection. *MTC*: medullary thyroid carcinoma.
